# Increased Hospitalization for IBD Patients Seen in the ER During the COVID-19 Pandemic

**DOI:** 10.1093/jcag/gwac020

**Published:** 2022-06-25

**Authors:** Gurpreet Malhi, Gurjot Minhas, Jason Chambers, Maria Mikail, Reena Khanna, Aze Wilson

**Affiliations:** Department of Medicine, Western University, London, Ontario, Canada; Department of Medicine, Western University, London, Ontario, Canada; Schulich School of Medicine, Western University, London, Ontario, Canada; Department of Medicine, Western University, London, Ontario, Canada; Division of Gastroenterology, Department of Medicine, Western University, London, Ontario, Canada; Division of Gastroenterology, Department of Medicine, Western University, London, Ontario, Canada; Division of Clinical Pharmacology, Department of Medicine, Western University, London, Ontario, Canada; Department of Physiology and Pharmacology, Western University, London, Ontario, Canada

**Keywords:** Acute care access, COVID-19, IBD

## Abstract

**Background:**

During the COVID-19 pandemic, the focus of many health care systems shifted in order to prioritize and allocate resources toward treating those affected by COVID-19. What this has meant for other patient populations remains unclear. We aimed to determine if there have been changes to acute care access for patients with inflammatory bowel disease (IBD) during the COVID-19 pandemic.

**Methods:**

A retrospective cohort study was performed in IBD patients seen during (March 1, 2020 to August 31, 2020) and before (March 1, 2019 to August 31, 2019) the COVID-19 pandemic. IBD-related emergency room (ER) access, hospitalization, inpatient care and follow-up and post-discharge ER access were assessed.

**Results:**

A total of 1229 participants were included. A higher proportion of patients accessed ER during the pandemic (44.6% versus 37.2%, *P* = 0.0097). A higher proportion of hospitalizations resulted from IBD-related ER visits during the pandemic period (41.6% versus 32.4%, OR = 1.48, 95% CI = 1.14 to 1.94, *P* = 0.0047), though length of stay was shorter (7.13 ± 8.95 days versus 10.11 ± 17.19 days, *P* = 0.015) and use of rescue infliximab was less. No change was seen in inpatient surgical intervention. Despite similar proportions of follow-up appointments post-hospital discharge (pre-pandemic, 77.9% versus pandemic, 78.3%), more ER visits occurred in the first 30 days following hospitalization for patients in the pandemic cohort (24.4% versus 11.1%, *P* = 0.0015).

**Conclusion:**

These data highlight the need for ER services and hospitalization amongst IBD patients during the COVID-19 pandemic. This suggests that a return to pre-pandemic IBD care infrastructure is needed to mitigate the need for acute care access.

## INTRODUCTION

The severe acute respiratory syndrome coronavirus 2 (SARS-CoV-2) outbreak, also known as the coronavirus disease of 2019 (COVID-19), initially began in a small cluster of patients in Wuhan, China. COVID-19 was declared a worldwide pandemic in March 2020 (1). As of July 2021, there were more than 180 million confirmed cases worldwide (2). Since the onset of the pandemic, the focus of many health care systems has shifted toward limiting non-essential interactions with health care providers and institutions in order to prioritize and allocate resources toward treating those affected by COVID-19 (1,3,4).

While the effect of COVID-19 has been felt amongst many patient populations (5,6), those with inflammatory bowel disease (IBD) may be particularly impacted. IBD is a relapsing-remitting autoimmune condition characterized by inflammation of the gastrointestinal tract that affects millions of people globally (4). Its chronicity, symptom overlap with other medical entities and need for long-term medical treatment necessitate ongoing medical surveillance and interaction with a host of health services at regular intervals. With the onset of the COVID-19 pandemic, many clinics and elective procedures were delayed, and in some cases, cancelled altogether. This included, but was not limited to, IBD follow-up appointments, endoscopy and ancillary services such as biomarker testing, all of which are deemed important for disease monitoring. It is unclear if the inability to fully access usual care, combined with patient-initiated delays due to the uncertainty and fear associated with a pandemic, resulted in more deleterious outcomes for patients with IBD (7–9).

As such, we aimed to evaluate the association between the early stages of the COVID-19 pandemic and the need to access emergency care, require in-hospital care and any delays in appropriate post-discharge follow-up for patients with IBD seen during the course of the COVID-19 pandemic compared to IBD patients seen before the pandemic.

## METHODS

### Subjects

A retrospective cohort study, using administrative data, was conducted in adult patients with IBD seen at three tertiary care centres affiliated with Western University, London, Ontario, Canada. Participants were assessed for eligibility based on their date of assessment and an IBD diagnosis established by the International Classification of Diseases, Tenth Revision (ICD-10) code and confirmed by manual review of the participant chart. Additionally, eligible participants were assessed in-person or virtually at a scheduled IBD clinic appointment by their treating gastroenterologist during at least one of two assessment periods (pre-pandemic, March 1, 2019 to August 31, 2019; during the pandemic, March 1, 2020 to August 31, 2020). Individuals with a planned admission to hospital for the purpose of surgery, an admission at the time of an outpatient clinic visit or transfer from a peripheral hospital due to a refractory IBD-related disease course were excluded from this cohort in addition to participants younger than 18 years of age.

The objective of the study was to characterize any differences in IBD emergency room (ER) care access, inpatient care and follow-up care and ER access post-hospitalization before and during the COVID-19 pandemic. Demographic data, including age, sex, disease type (one of ulcerative colitis [UC] or Crohn’s disease [CD]) and location, and postal code (where postal codes containing ‘0’ as the second character were deemed rural based on the Canada Post Corporation) were obtained. Other variables collected included number of and reason for ER visits, hospitalization and reason for hospitalization, biologic and/or glucocorticoid use at the time of hospitalization, length of stay, inpatient surgery and use of rescue infliximab in hospital. Moreover, time to and mode (telephone versus in-person) of post-discharge follow-up were collected. Participants were followed for up to 7-month post-discharge to assess whether or not outpatient post-discharge follow-up was received from their treating gastroenterologist.

The primary outcome of this study was the proportion of hospitalizations relative to ER visits. Secondary outcomes included the number of ER visits, the length of stay, the proportion of participants requiring rescue infliximab or surgical intervention, and the incidence of and time to follow-up, and post-discharge ER visits within 30 days.

### Ethical Approval

The study protocol was approved by the Western University Health Sciences Research Ethics Board (REB). All methods were performed in accordance with the relevant guidelines and regulations of the Tri-Council Policy Statement (REB number 116407).

### Statistical Analysis

All statistical analyses were performed using GraphPad Prism version 9 and R software version 4.0.3. A *P*-value of <0.05 was determined to be significant. Descriptive statistics were used to summarize differences between the cohorts. Continuous variables are presented as means with standard deviations or medians with interquartile ranges (IQR) and are assessed using the Student’s *t*-test. Categorical variables are presented as proportions and assessed using Fisher’s exact test. For the evaluation of length of hospitalization, a multivariable linear regression was conducted adjusting for age, sex, rurality and disease type. Similarly, for the evaluations of surgical risk, need for rescue inpatient infliximab, and occurrence of post-discharge follow-up, a multivariable logistic regression was conducted adjusting for age, sex, rurality and disease type. Lastly when examining the 30-day return to ER, a multivariable logistic regression was conducted adjusting for age, sex, rurality, disease type and year.

## RESULTS

**Figure 1.**
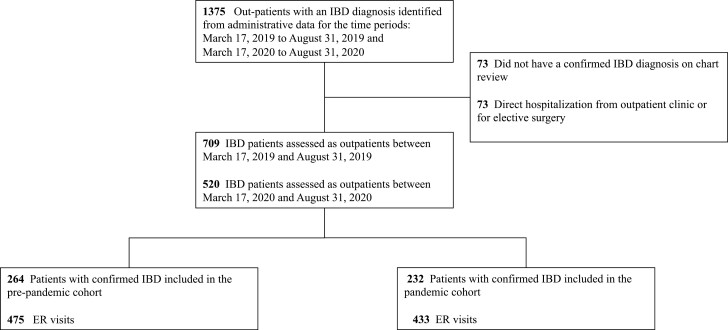
Study flow chart. ER, emergency room; IBD, inflammatory bowel disease.

Participant flow through the study and the baseline characteristics are highlighted in Figure 1 and Table 1, respectively. A total of 1375 patients with IBD were eligible for review based on the inclusion criteria. A total of 146 participants were excluded for not having an IBD diagnosis, being directly admitted to hospital from an outpatient clinic or having an elective admission for the purpose of a planned surgery (Figure 1). A total of 1229 patients with IBD were included of which 264 patients accessed ER care due to IBD pre-pandemic 475 times (pre-pandemic cohort) and 232 patients accessed ER care due to IBD during the early stages of the pandemic 433 times (pandemic cohort). Baseline characteristics were similar between cohorts. The mean length of follow-up was 32.36 ± 34.01 days for the pre-pandemic cohort and 30.51 ± 29.14 days for the pandemic cohort (*P* = 0.679).

**Table 1.** Baseline characteristics

Variable	Pre-pandemic cohort (*n* = 709)^*^	Pandemic cohort (*N* = 520)^**^	*P*-value
Mean age, years (range)	47.2(18–85)	46.9 (18–86)	0.65
Female sex (%)	354 (49.9)	252 (48.5)	0.64
Crohn’s disease (%)	437 (61.7)	311 (59.8)	0.55
Rural address (%)	164 (23.1)	118 (22.7)	0.89

Pre-pandemic cohort refers to participants seen between March 1, 2019 and August 31, 2019.

Pandemic cohort refers to participants seen between March 1, 2020 and August 31, 2020.

More IBD outpatients accessed ER services due to IBD during the pandemic period (pandemic cohort, *n* = 232/520, 44.6% versus pre-pandemic cohort, *n* = 264/709, 37.2%, *P* = 0.0097). A median number of 1 IBD-related ER visit per patient (IQR = 1) was observed pre-pandemic and during the pandemic. For the pre-pandemic cohort, 38.6% (*n* = 102/264) of participants had more than 1 IBD-related ER visit and 17.0% (*n* = 45/264) had 3 or more IBD-related ER visits. For the pandemic cohort, 41.4% (*n* = 96/232, *P* = 0.52) had more than one IBD-related ER visit and 17.2% (*n* = 40/232, *P* = 0.99) had 3 or more IBD-related ER visits.

For the pre-pandemic cohort, 154 hospitalizations (32.4%) resulted from 475 IBD-related ER visits. For the pandemic cohort, 180 hospitalizations (41.6%) resulted from 433 ER visits (*P* = 0.0047). The most frequent reason for admission to hospital was related to uncontrolled IBD-related disease activity (pre-pandemic cohort, 51.6%; pandemic cohort, 61.8%). Other reasons for admission included an IBD drug-related adverse event or infection. The mean length of hospital stay for the pre-pandemic cohort was longer, with a mean duration of 10.11 ± 17.19 days versus 7.13 ± 8.95 days for the pandemic cohort with adjustment for the covariates age, sex, rurality and disease type (adjusted *P* = 0.015) (Supplemental Table 1).

No difference was reported in the proportion of inpatient IBD-related surgeries between the pre-pandemic and pandemic cohorts with adjustment for the covariates age, sex, rurality and disease type. Thirty-five inpatient IBD-related surgeries took place amongst the pre-pandemic cohort who required hospitalization (22.7%) compared to 28 inpatient IBD-related surgeries amongst the hospitalized pandemic cohort (15.6%, adjusted *P* = 0.19). However, women were nearly twice as likely to progress to inpatient surgery while hospitalized compared to men (OR = 1.79, 95% CI = 1.02 to 3.13, *P* = 0.043) (Supplemental Table 2). Additionally, the average time to surgery was 5.3 ± 11.43 days for the pre-pandemic cohort and 5.19 ± 8.01 days for the pandemic cohort (*P* = 0.517).

For the pre-pandemic cohort, infliximab was administered during 20 hospitalizations out of 154 (13.0%) compared to 8 administrations out of 180 hospitalizations amongst the pandemic cohort (4.4%, OR = 3.03, 95% CI 1.23 to 8.33, *P* = 0.0098). This finding remained significant when adjusting for age, sex, rurality and disease type (3.45, 95% CI = 1.48 to 8.83, *P* = 0.0059). Individuals with UC were more likely to receive infliximab when hospitalized (OR = 2.94, 95% CI 1.31 to 6.89, adjusted *P* = 0.01) (Supplemental Table 3).

For the pre-pandemic cohort, 120 outpatient appointments were completed in follow-up to 154 hospitalizations (77.9%) compared to 141 outpatient follow-up appointments for 180 hospitalizations for the pandemic cohort (78.3%). There was no significant difference in the occurrence of post-discharge follow-up care pre-pandemic versus during the pandemic with adjustment for age sex, rurality and disease type (adjusted *P* = 0.29) (Supplemental Table 4). Interestingly, follow-up was less likely with increasing age (OR = 0.98, 95% CI = 0.97 to 0.99, *P* = 0.0027). There was a significant difference in how follow-up appointments were conducted for each cohort. Virtual assessment by phone was conducted for 8.3% (*n* = 10/154) and 72.3% (*n* = 102/141) for the pre-pandemic and pandemic cohorts, respectively (*P* < 0.0001). Lastly, in the pre-pandemic cohort, 17 new ER visits occurred within 30 days of hospital discharge out of 154 hospitalizations (11.1%) versus, in the pandemic cohort, 44 new ER visits occurred within 30 days of hospital discharge out of 180 hospitalizations (24.4%, *P* = 0.0015).

## DISCUSSION

The COVID-19 pandemic has impacted IBD patients by limiting health care delivery, including access to in-person assessments, endoscopies and elective surgeries (10,11). It is unknown if these changes to care delivery and access have resulted in deleterious outcomes for IBD patients, including the need for more emergency care, hospitalization or surgery. We aimed to assess the association between the period of the COVID-19 pandemic and the need to access emergency care, require in-hospital care and any delays in appropriate post-discharge follow-up for patients with IBD. Our study suggests that through the period of the COVID-19 pandemic, more IBD patients accessed ER services and a higher proportion of these patients required hospitalization when seen through the ER. Interestingly, the length of stay and use of inpatient rescue infliximab was less for patients seen within the pandemic period, while no difference was seen in the need and time to inpatient surgical intervention. Moreover, the proportion of patients seen in follow-up post-discharge was the same for both cohorts, though more patients were assessed by phone rather than in-person throughout the pandemic period. Lastly, a higher proportion of hospital discharges were associated with early re-presentation to the ER during the pandemic.

It is well documented that key measures put in place to mitigate the risk of the COVID-19 pandemic included transitioning from in-person care to virtual appointments, delaying or cancelling non-urgent procedures and reducing access to ancillary outpatient testing (12). We hypothesized that patients with IBD who are followed routinely by their family physician or gastroenterologist had reduced access to these care services due to the pandemic. This may account for the greater ER access seen during the pandemic. This opposes what has been reported with respect to ER access for all causes; a decrease in ER visits was a phenomenon seen across many countries through the COVID-19 pandemic. The Canadian Institute for Health Information (CIHI) reported that ER visits declined by 10% to 50% through periods of the pandemic in 2020 (13). The Centers for Disease Control and Prevention (CDC) reported declines by up to 42% in the United States and the National Health Service reported declines of up to 50% in the United Kingdom (14,15). It is speculated that the threshold for accessing emergency services was higher amongst individuals, leading some individuals to delay or avoid attending the ER due to concerns around SARS-CoV-2 exposure. Additionally, we report more ER presentations resulting in IBD-related hospitalizations for individuals seen during the pandemic period. This may relate to the delay and/or cancellation of key testing, such as outpatient investigations required for disease monitoring or outpatient endoscopy that typically provides the opportunity for early intervention. Despite an increase in hospitalizations relative to ER presentations in the pandemic period, fewer patients were given infliximab as rescue therapy in hospital, there was no difference in in-hospital surgical interventions, and overall hospital length of stay was shorter. This may reflect a more moderate population of IBD patients who simply required specific interventions such as intravenous glucocorticoids to achieve disease control and could be effectively discharged. Other reasons for these findings could relate to the COVID-19 pandemic, including an aversion for applying aggressive immunosuppressive therapies, reserving surgical access for the most urgent or emergent patients and minimizing infectious exposure risk by minimizing time in hospital.

Moreover, upon discharge, there was no difference in the occurrence of or time to post-hospitalization follow-up between the periods before and during the pandemic; however, there was a notable difference in the method of follow-up. During the pandemic period, more patients were followed up by telephone compared to the period before the pandemic, where the majority of follow-up occurred in person. These findings are in line with recommendations by various government agencies (16,17) and IBD organizations where virtual assessments were encouraged through the period of the COVID-19 pandemic (18).

Lastly, more return visits to the ER following hospital discharge were seen during the period of the pandemic. One reason for this may have related to increasing pressures to discharge patients to minimize COVID-19 infection risk. Alternatively, the use of virtual follow-up appointments would have resulted in an inability to physically examine patients and could have contributed to a lack of opportunity to identify sicker patients who needed earlier intervention.

Limitations of this study include its retrospective study design and its evaluation of IBD patients seen at only three tertiary care centres affiliated with a single university. There may be fundamental differences in IBD patients who attend community hospitals, including the complexity of IBD and their perceptions of the COVID-19 pandemic and associated precautions. Additionally, the study herein evaluated a limited segment of the COVID-19 pandemic. This pandemic continues to date, and thus the extent of its impact on the care of immunosuppressed patients may not be fully reflected herein.

Conversely, there has been limited, objective evaluation of what has happened to patients with IBD through the period of the COVID-19 pandemic and the effect of sweeping and rapid care changes. A strength of this study described here is the use of objective measures such as ER access, hospitalization and receipt of care follow-up to provide insight into how the COVID-19 pandemic may have additionally impacted IBD patients beyond the impact of primary infection. In other disciplines, this has been well documented. For example, in the setting of renal and liver transplantation, decreases in organ procurement, increases to post-transplant and waitlist mortality have been described throughout the period of the pandemic (19,20). The impact on the care of IBD patients has been largely limited to patient surveys that have subjectively documented the experience of IBD patients through the COVID-19 pandemic but the effect on objectives measures has been less well described (21,22).

## CONCLUSION

Ultimately, changes were seen in the access of acute care for IBD patients during the early stages of the COVID-19 pandemic. We report a greater use of ER services amongst IBD patients assessed as outpatients during the first waves of the pandemic as well as a greater need for hospitalization amongst ER-assessed patients; however, hospitals stays were shorter, with a decreased use of infliximab rescue therapy and no change in surgical intervention during hospitalization. Higher ER attendance was seen within the first 30 days of discharge in the pandemic cohort. This highlights the likely benefit of structured, in-person assessments and follow-up to mitigate the need for more acute care for this patient population. Future studies evaluating the long-term impact of the pandemic across all waves on IBD care and outcomes are needed. Given that the pandemic is ongoing, strategies to circumvent poor outcomes in patients are needed.

## Supplementary Material

gwac020_suppl_Supplementary_Material
