# Supplementary material for: Increased Hospitalization for IBD Patients Seen in the ER During the COVID-19 Pandemic
Source: J Can Assoc Gastroenterol. 2022 Jun 25;5(6):271–5. doi: 10.1093/jcag/gwac020 (PMC9278246; doi:10.1093/jcag/gwac020)
Supplement: gwac020_suppl_Supplementary_Material [file gwac020_suppl_supplementary_material.docx]

**Supplemental Materials: Increased hospitalization for IBD patients seen in the ER during the COVID-19 pandemic**

**Supplemental Tables**

Supplemental Table 1: Multiple linear regression model for the effect on length of stay for all hospitalized patients (n=334)

| **Variable** | **Estimate** | **95% CI** | **p-value** |
| --- | --- | --- | --- |
| **Year 2020** | **-3.437** | **-6.206 to -0.6678** | **0.0151** |
| Age | 0.008452 | -0.06786 to 0.08476 | 0.8277 |
| Male Sex | 1.403 | -1.388 to 4.194 | 0.3235 |
| Rural address | -0.1412 | -3.433 to 3.151 | 0.9328 |
| UC diagnosis | -1.151 | -4.047 to 1.746 | 0.4351 |

95% confidence interval, 95%CI; ulcerative colitis, UC

Supplemental Table 2: Multiple logistic regression for the effect on surgery for all hospitalized patients (n=334)

| Variable | Odds ratio | 95% CI | p-value |
| --- | --- | --- | --- |
| Intercept | 3.240 | 1.311 to 8.244 | 0.0119 |
| Year 2020 | 1.452 | 0.8349 to 2.543 | 0.1880 |
| Age | 1.008 | 0.9921 to 1.024 | 0.3455 |
| **Male sex** | **0.5622** | **0.3191 to 0.9794** | **0.0434** |
| Rural address | 1.137 | 0.5996 to 2.274 | 0.7036 |
| UC diagnosis | 1.490 | 0.8310 to 2.750 | 0.1896 |

95% confidence interval, 95%CI; ulcerative colitis, UC

Supplemental Table 3: Multiple logistic regression for the effect on the use of rescue infliximab during hospitalization for all hospitalized patients (n=334)

| Variable | Odds ratio | 95% CI | p-value |
| --- | --- | --- | --- |
| **Year 2020** | **0.2896** | **0.1133 to 0.6750** | **0.0059** |
| Age | 0.9917 | 0.9682 to 1.015 | 0.4832 |
| Male sex | 2.133 | 0.9489 to 5.053 | 0.0728 |
| Rural address | 1.554 | 0.6040 to 3.701 | 0.3347 |
| **UC diagnosis** | **2.939** | **1.305 to 6.887** | **0.0103** |

95% confidence interval, 95%CI; ulcerative colitis, UC

Supplemental Table 4: Multiple logistic regression for the effect on occurrence of post-hospitalization GI follow-up appointment for all hospitalized patients (n=334)

| Variable | Odds ratio | 95% CI | p-value |
| --- | --- | --- | --- |
| Year 2020 | 1.274 | 0.8110 to 2.006 | 0.2940 |
| **Age** | **0.9810** | **0.9686 to 0.9933** | **0.0027** |
| Male sex | 1.562 | 0.9884 to 2.488 | 0.0577 |
| Rural address | 0.9496 | 0.5589 to 1.642 | 0.8503 |
| UC diagnosis | 0.7262 | 0.4524 to 1.166 | 0.1844 |

95% confidence interval, 95%CI; ulcerative colitis, UC
